# Cerebellar Granulocytic Sarcoma: A Case Report

**DOI:** 10.5505/tjh.2012.65002

**Published:** 2012-06-15

**Authors:** Birol Baytan, Melike Sezgin Evim, Adalet Meral Güneş, Hasan Kocaeli, Şaduman Balaban, Ender Korfalı, Nükhet Tüzüner

**Affiliations:** 1 Uludağ University, School of Medicine, Department of Pediatric Hematology, Bursa, Turkey; 2 Uludağ University, School of Medicine, Department of Neurosurgery, Bursa, Turkey; 3 Uludağ University, School of Medicine, Department of Pathology, Bursa, Turkey; 4 İstanbul University, School of Medicine, Department of Pathology, Bursa, Turkey

**Keywords:** children, Acute leukemia, Granulocytic sarcoma

## Abstract

Granulocytic sarcoma is a rare tumor composed of immature granulocytic cells that is usually associated with acute myelogenous leukemia. Intraparenchymal cranial localization without skull, meningeal, or bone marrow invasion is extremely rare. The mechanisms of intraparenchymal cranial localization of GS remains unknown, as only 10 cases with cerebellar granulocytic sarcoma have been previously reported. Herein, we report a four year old boy with cerebellar localization of granulocytic sarcoma.

## Abstract (tr)

Granulositik sarkom, genellikle akut myelositer lösemiye eşlik eden immatür granülositik hücrelerden oluşan nadir bir tümördür. Kafatası, meninks ve kemik iliği tutulumu olmaksızın izole intrakranial parankimal tutulum ise çok daha nadirdir ve mekanizması henüz tam olarak bilinmemektedir. Literatürde 10 vaka bildirilmiştir. Bu raporumuzda kemik iliği tutulumu olmadan izole serebellar parankimde yer alan granülositik sarkom tartışılmıştır.

## INTRODUCTION

Granulocytic sarcoma (GS) is a rare extramedullary solid tumor. GS, which is synonymous with chloroma, myeloblastoma, and extramedullary leukemia, is a localized tumor composed of leukemic myeloblasts and myeloid precursors. The clinical characterization of GS is classified into 4 groups: a) primary GS; b) GS at onset of acute myelogenous leukemia (AML); c) GS as an isolated recurrence of AML (new isolated focus of GS that occurs during bone marrow remission); d) GS with concurrent bone marrow relapse of AML [1,2,3,4]. The precise incidence rate of GS is not known, although it occurs in approximately 3% of patients with leukemia [4,5]. The most common sites of involvement are bone, periosteum, soft tissue, lymph nodes, and skin; however, intraparenchymal involvement of GS in the central nervous system (CNS) is rare [6-8]. Some Turkish researchers have reported that there is an association between AML and GS [9,10,11,12]. Çavdar et al. being the first in 1971 [13]. Herein we present a patient with the unusual presentation of cerebellar parenchymal GS invasion without skull, meningeal, or bone marrow invasion after completing treatment for AML.

## CASE REPORTS

A 4-year-old boy was diagnosed as AML without CNS involvement in May 2008. Bone marrow aspirate showed diffuse infiltration of monoblasts (90%). Flow cytometric analysis showed that these blasts were positive for myeloperoxidase, CD15, CD33, CD13, CD14, and CD4. Based on all these findings, the diagnosis of AML, FAB subtype M5 was confirmed. Complete remission was achieved following 4 courses of the Medical Research Council (MRC) UK-AML 12 chemotherapy protocol (2. remission induction courses with cytarabine, daunorubicin, and etoposide [ADE] given for 10, 3, and 5 d, respectively, for the 1st course, and 8, 3, and 5 d, respectively, for the 2nd course, and 2 consolidation courses consisting of amsacrine, cytarabine, etoposide [MACE], and MidAC [mitoxantrone, cytarabine] and triple intrathecal chemotherapy given once after each chemotherapy courses [14]. Informed consent was obtained from patient’s family. 

**Figure 1:**
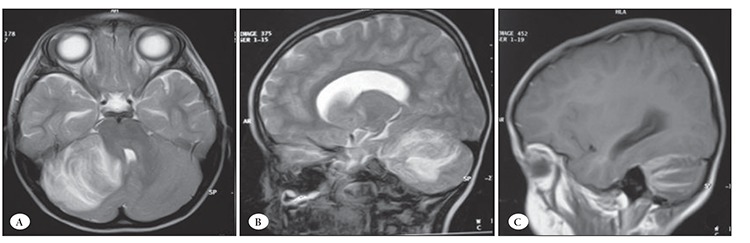
Axial (A) and sagittal (B-C) T1- and T2-weighted MRI with Gd-DTPA show the linear heterogeneously enhanced mass inthe right cerebellar hemisphere.

At the end of the re-consolidation phase of treatment the patient had frontal headache, but no fever or neurological findings. Cranial magnetic resonance imaging (MRI) performed due to the persistent headache showed a 4 x 4.5 x 5-cm mass lesion in the right cerebellar hemisphere. The lesion was hypointense on T1-weighted and hyperintense on T2-weighted MRI, and showed linear heterogeneous enhancement after administration of Gd-DTPA (Figure 1). Left facial weakness and a slight increase in deep tendon reflexes were noted within 2 hours after cranial MRI. Neurological examination showed that the patient had aphasia, hemiparesis, papilloedema, and 3rd (III), 7th (VII), and 9th (IX) cranial nerve palsies, and he was scheduled for surgery. 

**Figure 2:**
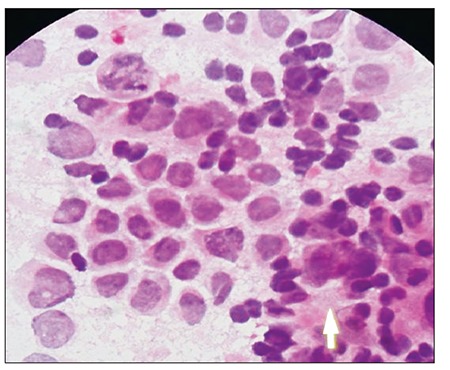
Photomicrograph shows diffuse undifferentiatedmononuclear cells (hematoxylin and eosin, 100x).

During preparation for surgery the patient’s clinical condition continued to deteriorate and he became comatose, with dilated non-reactive pupils and extensor posturing in response to painful stimuli. Because these findings suggested tonsillar herniation, the patient underwent emergency surgery and the mass was resected via right suboccipital craniotomy while in the sitting position. The mass was removed, with the exception of a small piece with a diameter of 1 cm that adhered to the brainstem and lower cranial nerves. Postoperatively the patient recovered in 2 days and regained consciousness and full physical strength. However the symptoms related to the 3rd , 7th and 9th cranial nerves improved nvolved retarded. Cerebrospinal fluid (CSF) obtained during the surgery was acellular. Both CSF protein (15 mg dL–1) and lactate dehydrogenase (14 IU L–1) levels were within the normal range. Histopathologic examination showed that the mass was composed of undifferentiated mononuclear cells with monoblastic features (Figure 2). Immunohistochemical staining exhibited weak myeloperoxidase positivity, and strong CD34 and CD117 monoclonal antibody positivity. The patient’s bone marrow aspiration done concurrently performed withwith cranial surgery was found in complete remission. 

Two weeks after surgery, the patient received 10 doses of cranial radiotherapy (RT) with a each dose of 1200 cGy with a each dose. The the symptoms related to involved cranial nerve paralysis had improved following the cranial RT. were. As the patient’s family refused systemic chemotherapy supportive therapy was initiated. Four week later the 10th dose of RT, the patient developed widespread polypus masses on the gingiva, and renal ultrasonography performed due to abdominal pain showed hypoechoic lesions in the renal parenchyma. These findings were indicative of the dissemination of leukemia. His bone marrow aspiration done in the same time with the dissemination of leukemia was also found infiltrated. The patient’s clinical condition progressively declined and he died 2 months after being diagnosed as cerebellar GS.

## DISCUSSION

The presented case developed cerebellar GS without CSF or bone marrow involvement. Intracranial GS without meningeal or skull invasion is extremely rare; only 10 pathologically proven cases have been reported [15]. There are 3 primary explanations of the pathogenesis of GS. The first is that the leukemic nodules arise from leukostatic plugging of small cerebral vessels during the acceleration of blastic phase [16]. The second is that leukemic cells passing through the first dura, and then the subarachnoid and Virchow-Robin spaces invade the brain surface [17]. The third is that the remainders of embryonic CNS cells capable of hematopoetic differentiation could undergo malignant transformation [18]. 

This presented case was morphologically classified as AML M5, based on flow cytometric observation of positive myeloid markers. The level of CD4 expression was high (98%). FAB subtype M4 and M5 morphology, and the presence of myeloblasts with high-level expression of CD4 were reported to be factors that predispose to the development of GS [15]. CSF samples obtained from the patient at the time of diagnosis and during chemotherapy were acellular, with normal protein and LDH levels. Furthermore, CSF obtained from the ventricles at the time GS was diagnosed was also acellular. Although only a small number of cases have been reported, approximately 35% of patients with intracranial GS had no CSF involvement [19,20]. 

Management of GS in the presented patient included surgical mass excision and RT. Data on GS are scarce and its optimal treatment is unclear; therefore, it should be considered a leukemic relapse site and treated as a relapse with intensive chemotherapy and RT, even though the bone marrow does not show any leukemic infiltration. The presented patient received 10 doses of cranial RT, but as his family refused systemic chemotherapy he was given only supportive therapy. Despite the fact that GS is very radiosensitive, time to remission is reported and most patients die within 3 months [21,22,23]. In the presented case, 1 month after the completion of RT, leukemia was disseminated simultaneously infiltrating the gingiva, renal parenchyma, and bone marrow, and the patient died due to disseminated disease after 2 months of the diagnosis of the cerebellar GS. 

## CONCLUSION

GS in brain parenchyma without skull or meningeal involvement is very rare. Its mechanism of formation is still unknown. Some degree of improvement can be achieved in such patients with surgery and RT; however, the prognosis remains poor. 

**Conflict of Interest Statement **

The authors of this paper have no conflicts of interest, including specific financial interests, relationships, and/ or affiliations relevant to the subject matter or materials included.
